# Comparative effectiveness of porcine placental extracellular matrix against other cellular, acellular and matrix-like products in diabetic foot ulcers from the Medicare database

**DOI:** 10.57264/cer-2025-0092

**Published:** 2025-07-25

**Authors:** Brad Marcinek, Jenny Levinson, Serena Nally, Irene Varghese, Caitlin Sheetz, Peter Kardel, Cristin Taylor

**Affiliations:** 1 Convatec Technology Centre, Lexington, MA 02421, USA; 2 ADVI Health LLC., Washington, DC 20004, USA

**Keywords:** CAMPs, cellular, acellular and matrix-like products, Center for Medicare & Medicaid Services, CMS, DFU, diabetes, diabetic foot ulcer, ECM, extracellular matrix, hard-to-heal wounds, health economics, Medicare, retrospective study, wound care

## Abstract

**Aim:**

Diabetic foot ulcers (DFUs) are often hard to heal and may require advanced treatment with cellular, acellular and matrix-like products (CAMPs). This retrospective cohort study examines the Medicare fee-for-service population to compare clinical outcomes and health resources utilization in patients receiving porcine placental extracellular matrix (PPECM [InnovaMatrix^®^ AC, Convatec Triad Life Sciences, LLC, TN, USA]) against other CAMPs.

**Materials & methods:**

The Center for Medicare & Medicaid Services 100% Research Identifiable Files database was searched for potentially eligible patients using relevant ICD-10 diagnosis codes between 1 January 2020 and 31 December 2023. Patients with confirmed diagnoses of DFU and non-pressure chronic ulcers were selected for further evaluation. Eligible patients were categorized into groups according to treatment received: PPECM, PPECM’s 510(k) predicate† (OASIS^®^ Wound Matrix, Cook Biotech Inc., IN, USA), or other similar CAMPs (OSC [Marigen Shield and Omega3 (Kerecis, Ísafjörður, Iceland); Integra Dermal Regeneration Template and Primatrix (Integra LifeSciences, NJ, USA); GraftJacket (Stryker, MI, USA); Theraskin and Dermacell (LifeNet Health, VA, USA); FlexHD/AllopatchHD and Amnioband (MTF Biologics, NJ, USA); Grafix/Stravix (Smith + Nephew, MA, USA); Epicord and Epifix (MiMedx, GA, USA); Affinity, Apligraf and Dermagraft (Organogenesis, MA, USA)]). Patient demographics, comorbidities and ulcer location were assessed for cohort homogeneity and balanced via inverse probability of treatment weighting, to adjust for potential confounding factors when comparing health outcomes.

**Results:**

There were 940,910 patients with a DFU diagnosis; of these, 738,124 (78.4%) also had same-day diagnosis of a non-pressure chronic ulcer. After application of eligibility criteria, there were a total of 34,664 patients with DFUs with 37,380 episodes of care that included one of the CAMP treatments (PPECM: number of patients (n) = 186, number of episodes (e) = 186; PPECM’s 510(k) predicate: n = 369, e = 370; and OSC: n = 33,858, e = 36,559). In the inverse probability of treatment weighting-balanced model, patients with OSC episodes were 1.309-times more likely to undergo outpatient amputation than those with PPECM (95% CI: 1.251–1.371, p < 0.0001). Bacteremia was 2.75-times more likely in OSC episodes than in PPECM episodes (95% CI: 2.471–3.053, p < 0.0001). PPECM episodes had significantly fewer outpatient hospital visits compared with both OSC and PPECM’s 510(k) predicate (5.84, 8.79 and 10.24, respectively, p < 0.0001). There were no differences in physician office visits or hospital admissions.

**Conclusion:**

The findings suggest that PPECM performed clinically as well as or better than other well-established CAMPs. Notably, episodes of DFU care that involved PPECM were associated with significantly fewer outpatient amputations, fewer episodes of bacteremia, and fewer outpatient hospital visits. These data suggest that PPECM offers a clinically competitive treatment option for patients with DFUs.

## Plain language summary

**What is this article about?:**

Diabetic foot ulcers are wounds that are often difficult to heal and pose significant health risks to patients, including infection, amputation and hospitalization, as well as economic and societal costs.This study examines the comparative effectiveness of porcine placental extracellular matrix against other well-established cellular, acellular and matrix-like products in the management of diabetic foot ulcers within the Medicare fee-for-service patient population.

**What were the results?:**

Patients with diabetic foot ulcers who received porcine placental extracellular matrix (PPECM) showed significantly lower relative risk for major and minor outpatient amputations and wound complications.Patients receiving PPECM also showed fewer outpatient hospital visits compared with similar cellular, acellular and matrix-like products and PPECM’s 510(k) predicate.

**What do the results mean?:**

Porcine placental extracellular matrix demonstrated lower healthcare utilization across all settings and clinical performance that surpassed or was comparable to that of other cellular, acellular and matrix-like products (CAMPs). This study builds on previous CAMPs literature, supporting evidence that have shown CAMPs application is associated with lower rates of amputation, a direct predictor of mortality rates in diabetic foot ulcers patients.

## Shareable abstract

Among Medicare patients with diabetic foot ulcers that were treated using cellular, acellular and matrix-like products, those receiving porcine placental extracellular matrix were observed to have lower rates of outpatient amputation and bacteremia than those treated with other products. #DFU #MedicareData #WoundCare #HealthUtilization

Diabetic foot ulcers (DFUs) are a common yet serious complication of diabetes, affecting as many as a third of people with diabetes over their lifetime [1]. Analysis of real-world data from the US Wound Registry provides compelling evidence that most DFUs do not heal (55–70%) [2]. This may be a consequence of the significant morbidity and mortality associated with DFUs, with up to 60% becoming infected and nearly 20% of infections leading to lower-extremity amputation (LEA) [3]. Within 5 years of DFU diagnosis, the overall mortality rate approaches nearly 50%, primarily due to cardiovascular issues and infections [4]; but after a major amputation, the 5-year mortality rate increases significantly, as high as 90% by some estimates [5].

Although historical data from preceding decades has shown a significant decline in the rates of diabetes-related LEAs, more recent data signal a slowing or even a reversal of this trend. An analysis of surveillance data from the US National Inpatient sample from 2009 to 2015 suggests that the rate of LEAs has rebounded, with the largest increase observed in younger and middle-aged adults with diabetes [6]. The upward trend of LEA rates has also been identified for older adults in an analysis of US Medicare fee-for-service (FFS) data, though the increase is less severe than in other age groups [7].

Beyond the increased risk of morbidity and mortality, DFUs impose significant human costs by restricting an affected individual’s productivity and employment opportunities, often leading to economic stress and emotional suffering [8]. Furthermore, DFUs impose significant societal and economic burdens. In the US, treatment expenses for the most severe ulcers can reach upwards of $50,000 per wound episode [1], and when amputation is necessary, the costs are nearly double [9–12]. Since ulcer duration has been shown to be an independent risk factor for infection [13–15], as well as for LEA [16–18], there is significant human, clinical and economic value in products that may decrease the time-to-wound closure and/or increase the incidence of complete closure.

Cellular, acellular and matrix-like products, or CAMPs, constitute a diverse class of therapeutic options for wounds that do not improve despite application of standard treatment techniques. The precise mechanism of action for any given CAMP will vary according to the source material used in its production [19], which may include biomaterials derived from animal or human tissues, or biocompatible synthetic materials. Porcine placental extracellular matrix (PPECM, InnovaMatrix^®^ AC) is the only CAMP on the market that is derived from porcine placenta and indicated for management of a wide array of challenging wound types, including DFUs.

As a class, CAMPs have been shown to be more effective than standard wound care alone in achieving complete wound closure in patients with DFUs [20,21]. For PPECM specifically, comparative studies of PPECM against standard of care (SOC) alone are in progress; however, a recent real-world study reported encouraging clinical performance of PPECM [22]. In this study PPECM was used in the treatment of 60 wounds, 52% of which were documented as limb/life threatening. PPECM showed encouraging results in this challenging population, achieving 53.3% wound closure at a median of 53 days.

There is a general absence of robust comparative evidence assessing different CAMPs in head-to-head randomized trials, cost–effectiveness analyses, and in real-world studies, all of which could aid decisions on which CAMP to use in clinical practice [21]. Regarding comparative health economics and outcomes research, the Medicare FFS claims database has been used previously to show that CAMPs were associated with significant reductions in major and minor amputation, emergency department visits and hospital readmissions as compared with the SOC cohort [23,24].

This study aims to assess the clinical outcomes associated with PPECM compared with other CAMPs on the market using the latest Medicare data, in an effort to close the gaps in comparative CAMPs evidence in the real-world treatment of DFUs.

## Materials & methods

### Data source & study design

This retrospective cohort study was conducted to examine the clinical outcomes and health resource utilization in patients receiving PPECM compared with other CAMPs. Data were obtained from the Center for Medicare & Medicaid Services (CMS) 100% Research Identifiable Files (RIFs) database. The dataset comprises administrative claims data from Medicare FFS beneficiaries enrolled in part A and part B coverage, including physician offices, outpatient hospitals, inpatient admissions, intensive care unit (ICU) visits, durable medical equipment (DME), home health, skilled nursing facilities and hospice care. The study utilized healthcare claims data from 1 January 2020 through 30 June 2024. The demographic characteristics of the study population were derived from the Master Beneficiary Summary File provided by the Chronic Conditions Data Warehouse. Patients with incomplete demographics or claims data were excluded from the study.

### Patient population selection

Patients with diagnoses of DFU and non-pressure chronic ulcers with at least one medical claim in Medicare Parts A and B were selected from the index period between 1 April 2020 and 31 December 2023.The pre-index period covered 90 days prior to the first diagnosis date, with data available from as early as 1 January 2020. The post-index period extended through 30 June 2024, allowing up to 6 months of follow-up after the latest claim date. The International Statistical Classification of Disease and Related Health Problems (ICD-10-CM) codes were used to identify patients with study relevant diagnoses (Table 1).

**Table 1.** Relevant ICD-10-CM codes used in cohort identification.

ICD-10-CM code	Diagnosis description
E08.621	Diabetes mellitus due to underlying condition with foot ulcer
E08.622	Diabetes mellitus due to underlying condition with other skin ulcer
E09.621	Drug or chemical induced diabetes mellitus with foot ulcer
E09.622	Drug or chemical induced diabetes mellitus with other skin ulcer
E10.621	Type 1 diabetes mellitus with foot ulcer
E10.622	Type 1 diabetes mellitus with other skin ulcer
E11.621	Type 2 diabetes mellitus with foot ulcer
E11.622	Type 2 diabetes mellitus with other skin ulcer
E13.621	Other specified diabetes mellitus with foot ulcer
E13.622	Other specified diabetes mellitus with other skin ulcer
L97.xxx	Non-pressure chronic ulcer of lower limb (including all child codes)

For consideration of eligibility, patients were required to have at least one claim associated with a dual diagnosis of both diabetic foot ulcer and non-pressure chronic ulcer of lower limb.

There were no restrictions on code ordering (e.g., E08.621 + L97.103 is equivalent to L97.103 + E08.621).

Following their first diagnosis date, patients were evaluated for their receipt of CAMPs reported under relevant Current Procedural Terminology codes for the application of CAMPs (15271, 15272, 15275, 15276, C5271, C5272, C5275 and C5276) and product-specific Healthcare Common Coding System (HCPCS) Q or A codes (Table 2).

**Table 2.** Definition of treatment groups by product-specific Healthcare Common Coding System codes.

Treatment group	Product name	HCPCS code
PPECM	InnovaMatrix AC	A2001
PPECM’s 510(k) predicate	Oasis Wound Matrix	Q4102
	Oasis Tri-layer Wound Matrix	Q4124
OSC	Kerecis Marigen/Shield	A2019
	Apligraf	Q4101
	Integra DRT	Q4105
	Dermagraft	Q4106
	Graftjacket	Q4107
	Primatrix	Q4110
	Theraskin	Q4121
	Dermacell	Q4122
	Flex HD/Allopatch HD	Q4128
	Grafix/Stravix	Q4133
	Amnioband	Q4151
	Kerecis Omega3	Q4158
	Affinity	Q4159
	Epifix	Q4186
	Epicord	Q4187
	Derma-Gide	Q4203

CAMP: Cellular, acellular and matrix-like products; HCPCS: Healthcare Common Coding System; OSC: Other similar CAMPs; PPECM: Porcine placental extracellular matrix.

A treatment episode included all relevant claims for CAMPs, spanning from the date of the first application to the date of the last treatment claim. A new episode was initiated if a gap of 90 days or more was observed between CAMPs. Episodes were included if they had at least one claim with the relevant HCPCS code (Table 2).

To ensure specificity in the care of the wounds and to guarantee a complete 6-month post-episode period, patients were excluded from the analysis if any of the following were present:

- Claim of non-pressure chronic ulcer in the 90-day period prior to their first diagnosis date.
- Claim of wound depth at the bone (ICD-10-CM: L97.xx4, L97.xx6).
- Received multiple treatment types across episodes.
- Treated with multiple CAMPs within an episode of care.
- Episodes concluded after 31 December 2023.
- Did not maintain continuous enrollment in Medicare Parts A, B and D from 90 days before their first diagnosis date to 6 months after their last episode date.

After inclusion and exclusion criteria were applied, patient episodes were divided into one of three treatment groups (Table 2).

### Study measures

Baseline characteristics, including demographics and the Charlson Comorbidity Index (CCI) were assessed to understand the differences between the treatment groups. Patient demographics measured at the first diagnosis date included age, gender, race, dual enrollment in Medicare and Medicaid, current reason for Medicare entitlement, and comorbidities. The total CCI score was calculated by summing the values of the 19 CCI comorbidities. All covariates were evaluated for differences to ensure accurate comparisons between treatment groups. The covariates used for balancing treatment groups included age, race, dual eligibility in Medicare and Medicaid, current reason for enrollment and congestive heart failure.

### Outcomes

The primary objective of this study was to comparatively evaluate the clinical effectiveness of CAMPs, by investigating rate of amputations, the use of adjunctive wound therapy and frequency of wound complications. The rate of amputations (in both inpatient and outpatient settings) and wound complications (such as bacteremia, cellulitis, lymphangitis, abscess, gangrene and sepsis) were assessed in the 6-month post-episode period. Episodes were also reviewed for adjunctive wound care treatments commonly used in the management of DFUs such as surgical (operating room) debridement, total contact casting, compression therapy, negative pressure wound therapy, hyperbaric oxygen therapy, MIST therapy and other adjunctive therapies. Healthcare resource utilization and Medicare reimbursement amounts were evaluated across various service sites, including physician offices, outpatient hospitals, inpatient admissions, ICU visits, DME, home health, skilled nursing facilities and hospice care.

### Statistical analyses

Descriptive statistics were used to summarize the demographics and baseline characteristics of the study population. Categorical variables were reported using percentages and continuous variables were reported using mean and standard deviation. Comparisons were made of both categorical and continuous variables, using chi-square and *t*-test, respectively. Differences in treatment groups were presented in terms of p-values and standard mean differences (SMD). Due to the correlation between p-values and population size, the SMD was used to determine imbalance, with SMD ≥10 indicating an imbalanced variable between groups. Inverse probability of treatment weighting (IPTW) was performed to ensure balance in characteristics and control for differences between treatment groups. To assess differences between groups after IPTW, weighted chi-square tests were performed to assess categorical variables for independence. For continuous variables, weighted *t*-tests were employed to evaluate differences in means between the groups. Regression models were used to further evaluate outcomes and control data skewness. Poisson regression models were used for patient visits. IPTW-adjusted Kaplan–Meier and Cox-Hazard regression analysis was performed to assess amputation-free survival in the 6 months post-episode period. All analyses were completed using SAS software v.9.4 (SAS Institute Inc, NC, USA).

## Results

**Figure 1.**
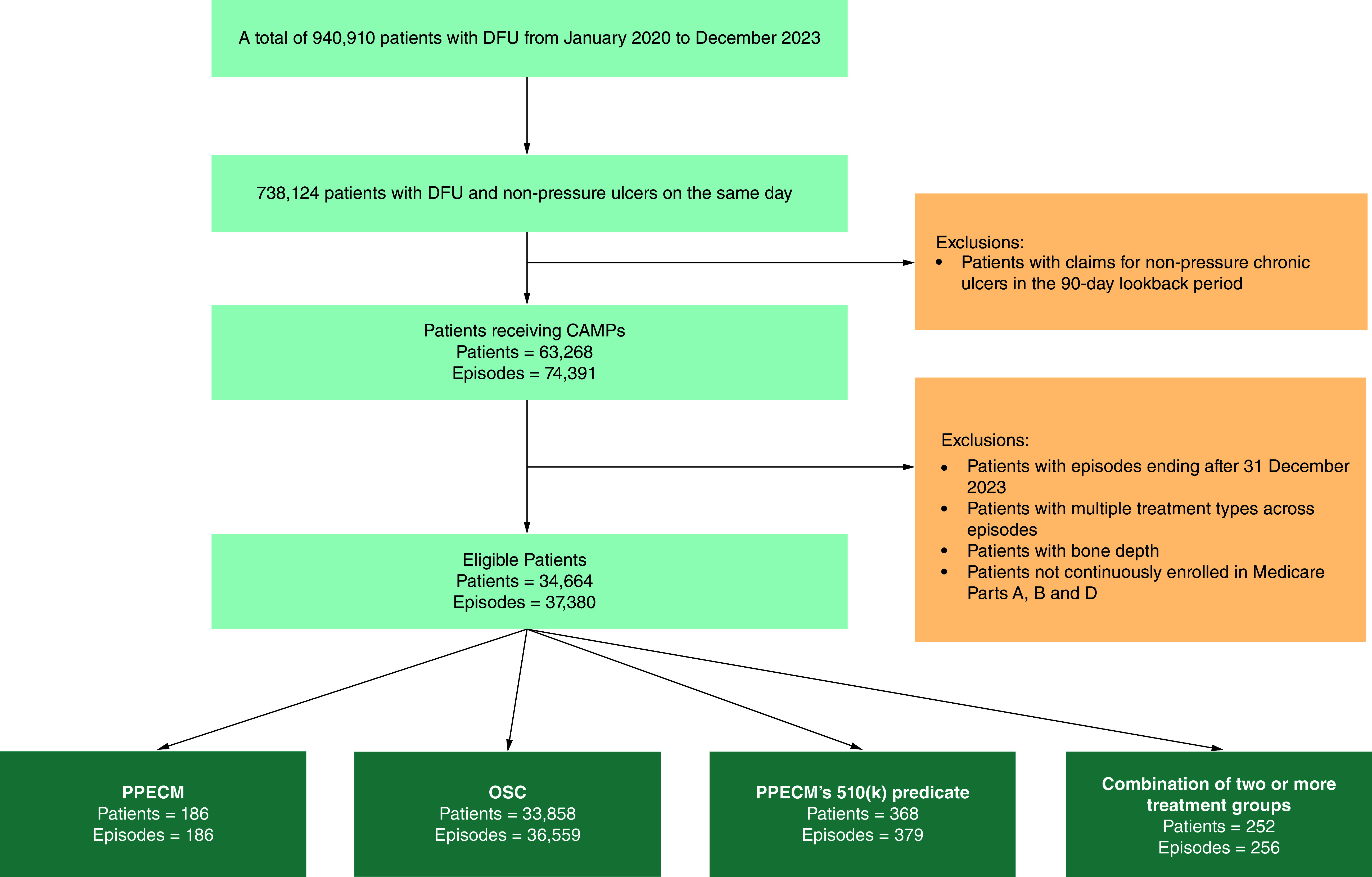
Stepwise attrition to identify eligible patients and episodes. CAMP: Cellular, acellular and matrix-like products; DFU: Diabetic foot ulcer; OSC: Other similar CAMPs; PPECM: Porcine placental extracellular matrix.

From 2020 to 2023, a total of 940,910 patients in the Medicare FFS population had a confirmed diagnosis of a DFU. Of these, 738,124 patients (78.4%) had diagnoses of both a DFU and a non-pressure chronic ulcer on the same day. After inclusion and exclusion criteria were applied (Figure 1 for summary), the study dataset included 37,380 patient episodes; 186 episodes (0.5%) were treated with PPECM, 36,559 episodes received other similar CAMPs (OSC) (97.8%) and 379 episodes (1%) were treated with PPECM’s 510(k) predicate. Additionally, 256 episodes (0.7%) involved a combination of at least two treatment groups, and were excluded from the analysis.

### Patient episode characteristics

The unweighted baseline demographic characteristics of the study population were striated for each treatment group (Table 3). Across the groups, the mean age ranged from 69.9 years in the OSC group to 72.1 years in the PPECM group. The distribution of age groups showed a similarly higher proportion of individuals aged 65–74 in all groups, with the OSC group having the highest percentage (41%) in this age bracket. The gender distribution across all treatment groups remained relatively consistent, with males accounting for 62–66% and females for 34–38%. The mean CCI ranged from 3.8 to 4.2 between the groups, with the OSC group having the highest CCI score (4.22, p = 0.001). The prevalence of peripheral vascular disease ranged from 56 to 62%, with the lowest proportion observed in the PPECM group and the highest in the OSC group. Diabetes without complications and diabetes with complications had similar presentations between the treatment groups. Nearly all patients (99–100%) had diabetes without complications; however, the prevalence of diabetes with complications ranged from 84 to 88% across groups. The proportion of renal disease was the same for the PPECM and the PPECM’s 510(k) predicate group (45%), with the OSC group having a slightly higher proportion (50%).

**Table 3.** Episode characteristics at baseline for all treatment groups.

Variable	Unweighted cohort					Weighted cohort				
	PPECM n (%)	OSC n (%)	SMD between PPECM and OSC	PPECM’s 510(k) predicate	SMD between PPECM and PPECM’s 510(k) predicate	PPECM (%)	OSC (%)	SMD between PPECM and OSC	PPECM’s 510(k) predicate	SMD between PPECM and PPECM’s 510(k) predicate
**Age**										
Mean age, years (st. dev.)	72.05 ± 11.28	69.94 ± 11.45	18.62	70.39 ± 11.77	14.45	70.03	69.96	0.08	70.05	0.02
< Age 65	35 (19%)	8,937 (24%)	13.69	92 (24%)	13.28	25%	24%	0.36	25%	0.19
Age 65–74	70 (38%)	14,907 (41%)	6.43	152 (40%)	5.06	40%	41%	0.12	40%	0.09
Age 75–84	58 (31%)	9,597 (26%)	10.90	90 (24%)	16.68	26%	26%	0.19	26%	0.07
Age >= 85	23 (12%)	3,118 (9%)	12.55	45 (12%)	1.51	9%	9%	0.05	9%	0.33
**Gender**										
Male	121 (65%)	24,137 (66%)	2.04	235 (62%)	6.32	66%	66%	0.15	62%	0.89
Female	65 (35%)	12,422 (34%)	2.04	144 (38%)	6.32	35%	34%	0.15	38%	0.89
**Race**										
White	141 (76%)	28,834 (79%)	7.31	297 (78%)	6.08	81%	79%	0.59	78%	0.72
Black	36 (19%)	4,106 (11%)	22.68	43 (11%)	22.31	10%	11%	0.42	12%	0.62
Asian	0 (0%)	397 (1%)		* (*)		0%	1%		1%	
Hispanic	* (*)	1,654 (5%)	16.93	18 (5%)	17.91	5%	5%	0.22	5%	0.19
Other	* (*)	1,568 (4%)	5.59	* (*)	7.78	4%	4%	0.03	4%	0.06
**Dual eligibility in Medicare and Medicaid**										
Dual	44 (24%)	14,122 (39%)	32.72	134 (35%)	25.82	39%	39%	0.14	38%	0.15
Not dual	142 (76%)	22,437 (61%)	32.72	245 (65%)	25.82	61%	62%	0.14	62%	0.15
**Reason for Medicare enrollment**										
Aged	148 (80%)	27,451 (75%)	10.71	286 (76%)	9.83	73%	75%	0.65	75%	0.49
Disability	* (*)	7,829 (21%)	12.10	* (*)	14.54	23%	21%	0.59	23%	0.10
ESRD	* (*)	1,279 (4%)	1.42	* (*)	9.78	4%	4%	0.23	2%	0.95
**Comorbidities**										
Mean CCI (st. dev.)	3.81 ± 1.60	4.22 ± 1.70	24.45	4.15 ± 1.70	20.55	3.95	4.21	2.27	4.22	1.90
Myocardial infarction	22 (12%)	4,828 (13%)	4.16	48 (13%)	2.55	14%	13%	0.21	13%	0.16
CHF	60 (32%)	13,738 (38%)	11.16	150 (40%)	15.27	35%	38%	0.76	40%	1.25
Peripheral vascular disease	104 (56%)	22,560 (62%)	11.78	226 (60%)	7.52	55%	62%	1.96	59%	1.02
Cerebrovascular	26 (14%)	5,512 (15%)	3.11	54 (14%)	0.77	15%	15%	0.03	14%	0.27
Dementia	* (*)	2,220 (6%)	7.99	26 (7%)	11.15	3%	6%	2.13	6%	1.67
COPD	21 (11%)	8,591 (24%)	32.60	91 (24%)	33.78	13%	24%	4.78	25%	3.95
Connective tissue disease	* (*)	1,903 (5%)	4.25	14 (4%)	3.09	5%	5%	0.54	4%	0.44
Peptic ulcer disease	0 (0%)	526 (1%)	n/a	* (*)	n/a	0%	1%	n/a	2%	n/a
Liver disease	* (*)	2,226 (6%)	5.50	27 (7%)	9.63	6%	6%	0.22	8%	0.56
Diabetes without complications	186 (100%)	36,437 (100%)	8.18	377 (99%)	10.29	100%	100%	11.98	100%	1.50
Diabetes with complications	157 (84%)	32,118 (88%)	9.96	326 (86%)	4.52	88%	88%	0.05	88%	0.01
Paraplegia and hemiplegia	* (*)	1,001 (3%)	0.31	13 (3%)	4.30	2%	3%	0.65	3%	0.75
Renal disease	83 (45%)	18,141 (50%)	10.01	169 (45%)	0.07	48%	50%	0.47	49%	0.35
Cancer	17 (9%)	3,276 (9%)	0.62	39 (10%)	3.88	9%	9%	0.07	9%	0.05
Moderate or severe liver disease	* (*)	306 (1%)	3.62	* (*)	0.14	1%	1%	0.21	1%	0.16
Metastatic carcinoma	* (*)	520 (1%)	3.12	* (*)	9.94	1%	1%	0.00	1%	0.46
HIV/AIDS	0 (0%)	181 (1%)	n/a	* (*)	n/a	0%	1%	n/a	1%	n/a

*Given datapoint comprised fewer than 11 patients and has been obscured in accordance with Center for Medicare & Medicaid Services Data Use Agreement requirements.

Values are presented as mean ± standard deviation or number (%). SMD ≥10 indicates an unbalanced cohort.

CCI: Charlson comorbidity index; CHF: Congestive heart failure; COPD: Chronic obstructive pulmonary disease; ESRD: End-stage renal disease; OSC: Other similar CAMPs; PPECM: Porcine placental extracellular matrix; SMD: Standard mean difference; St. dev.: Standard deviation.

### Rate of usage of adjunctive wound care treatments in combination with CAMPs during treatment episode

**Figure 2.**
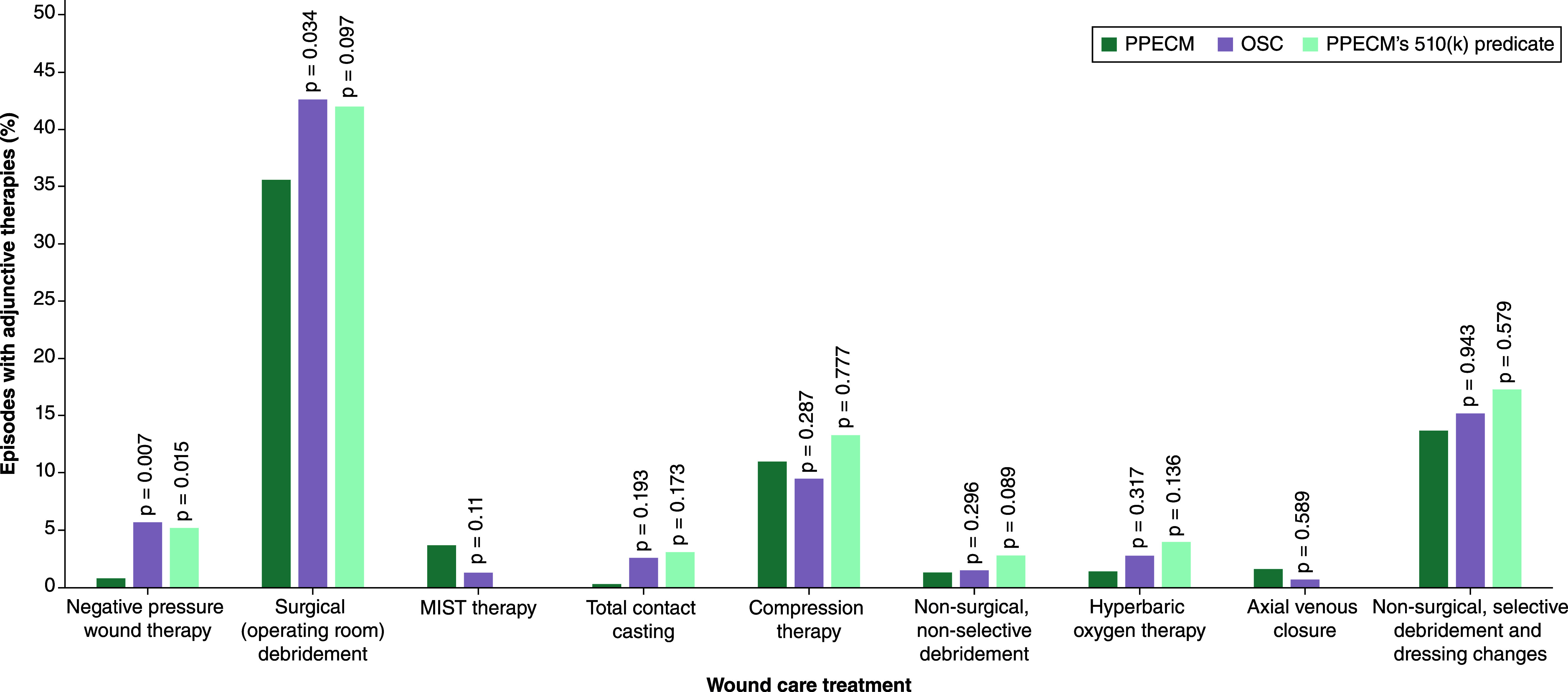
Rate of adjunctive therapies used in combination with cellular, acellular and matrix-like products during the patient episode. A p-value less than <0.05 indicates statistical significance. OSC: Other similar CAMPs; PPECM: Porcine placental extracellular matrix.

To evaluate treatment patterns during the episodes, the rate of adjunctive therapies was evaluated in Medicare Parts A and B (Figure 2). The rate of surgical (operating room) debridement use was lower in the PPECM group (36%, p = 0.034) compared with the OSC group (43%) and the rate of negative pressure wound therapy use was lower in the PPECM group (1%, p = 0.007) compared with the other treatment groups (5–6%). Other therapies were evaluated but did not show a significant difference in the usage rates across groups.

### Wound complications in the 6-month post-episode period

**Figure 3.**
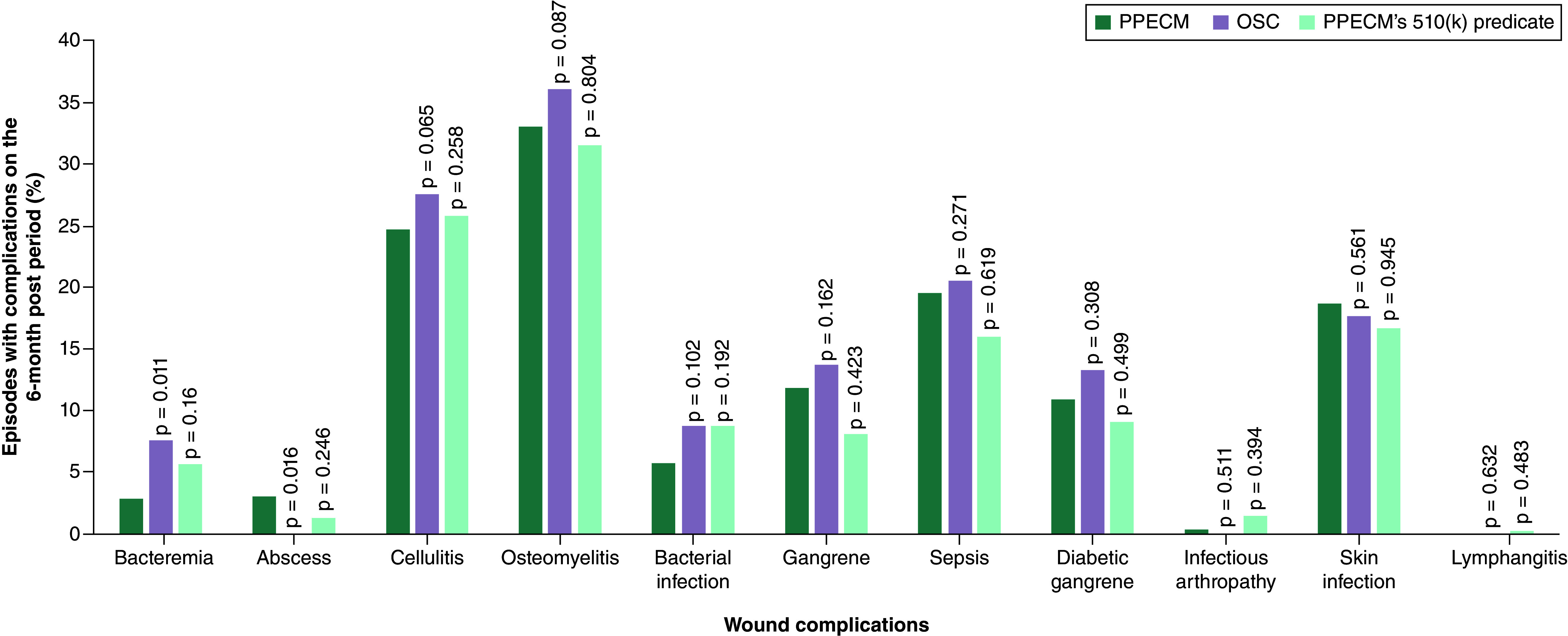
Wound complications in the 6-month post-episode period. A p-value less than <0.05 indicates statistical significance. OSC: Other similar CAMPs; PPECM: Porcine placental extracellular matrix.

The overall rate of wound complications was assessed in the post-episode period. No significant difference was observed in the total or individual wound complications rates when comparing PPECM to PPECM’s 510(k) predicate. However, significant differences were noted between the PPECM and OSC groups. Across all complications evaluated, the PPECM group (57%, p = 0.034) had a lower proportion of patients with wound complications compared with the OSC group (60%). Bacteremia was 2.75-times more likely in OSC group (95% CI: 2.471–3.053, p < 0.0001) and 1.99-times more likely in the PPECM’s 510(k) predicate group compared with PPECM. Abscess was more common in the PPECM group compared with OSC group (Figure 3).

### Amputations in the 6-month post-episode period

The rate of major and minor amputations was assessed in inpatient, outpatient, and across both settings combined. It should be noted that patients receiving inpatient and outpatient amputations were not mutually exclusive groups, as a patient could have multiple amputations in the post-episode period. After application of IPTW, the PPECM group showed no significant differences with the PPECM’s 510(k) predicate groups in amputations. The overall and inpatient amputation rates did not significantly differ between PPECM and OSC; however, outpatient amputations were more frequent in the OSC group (35%) than in the PPECM group (29%) with a borderline significant difference (p = 0.058).

**Figure 4.**
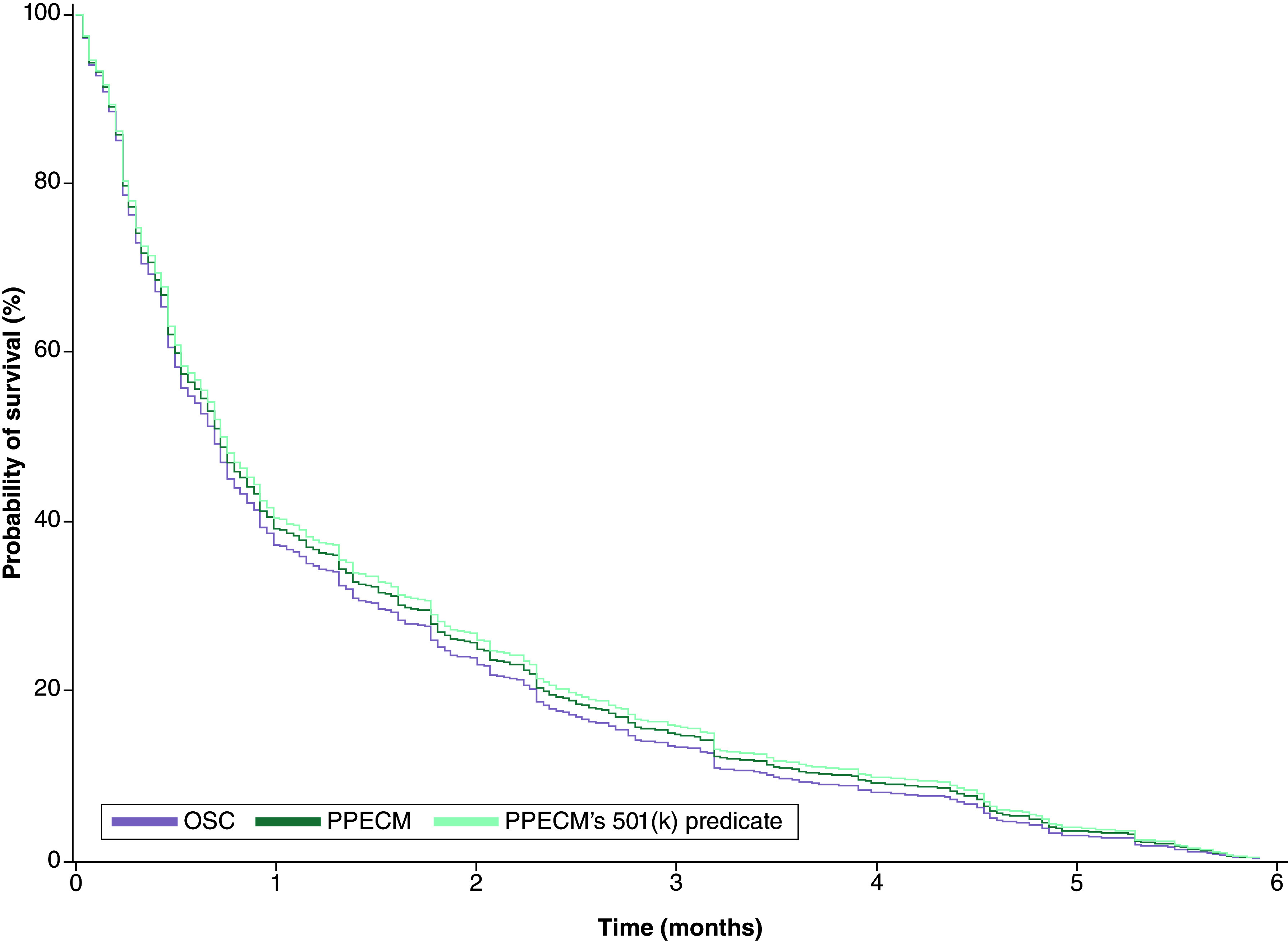
Inverse probability of treatment weighting-adjusted Kaplan–Meier curves for amputation-free survival for patients in the treatment groups over the 6-month post-episode period. OSC: Other similar CAMPs; PPECM: Porcine placental extracellular matrix.

A logistic regression predicting outpatient amputation by treatment grouping was performed to examine the borderline results further. The analysis found that the OSC group was 30.9% more likely to have an outpatient amputation compared with the PPECM group (odds ratio [OR] = 1.309, CI: 1.251–1.371, p < 0.0001). Additionally, a Cox-hazard analysis on the time to first amputation in the 6-month post-episode period found a 5.8% higher relative risk of amputation for patients in the OSC group (HR: 1.058, CI: 1.022–1.095, p = 0.002) compared with those in the PPECM group. No significant differences were observed between the PPECM and the PPECM’s 510(k) predicate group (p = 0.176) (Figure 4).

### Healthcare resource utilization in the 6-month post-episode period

**Figure 5.**
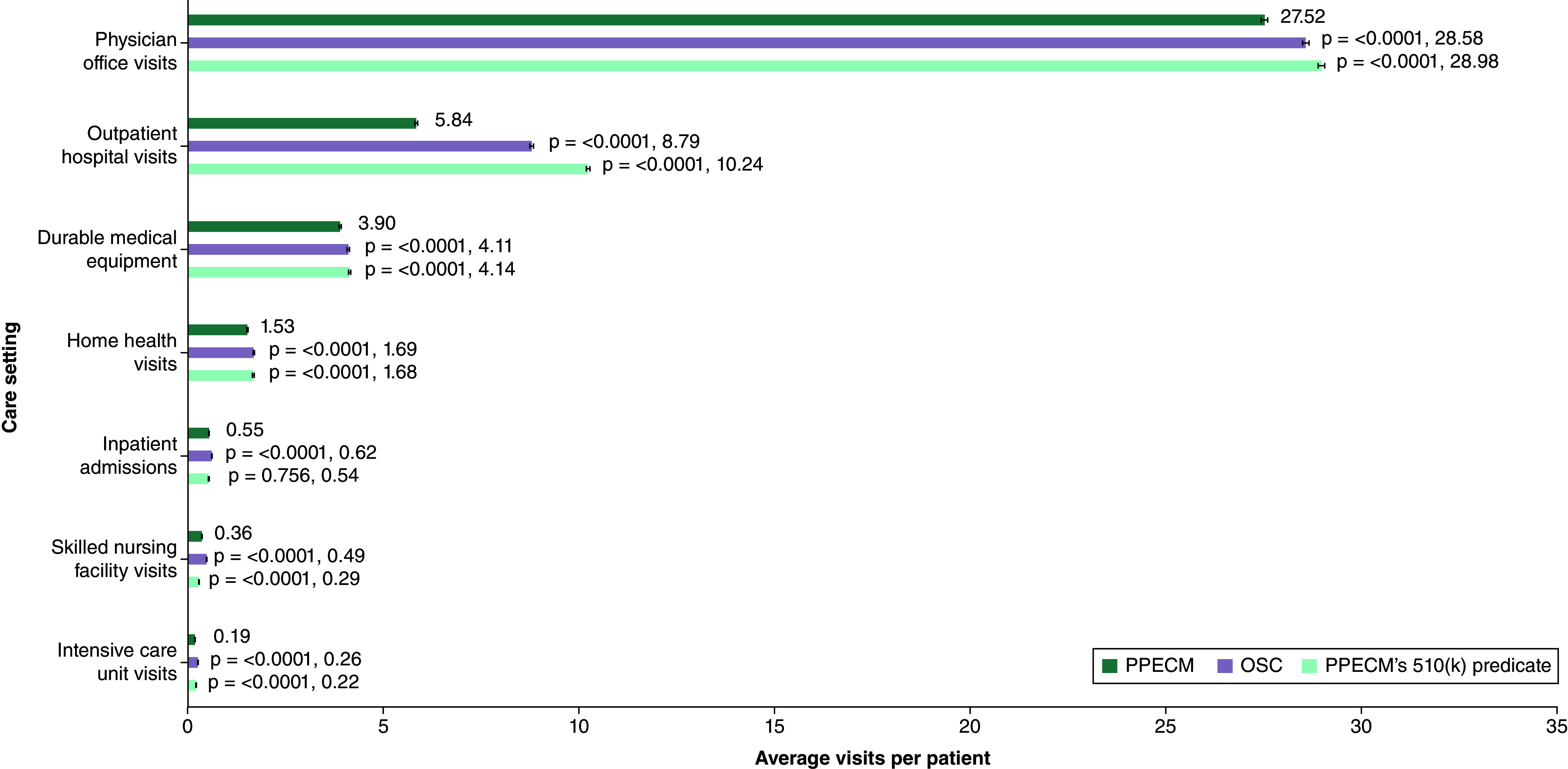
Average visits per patient in the 6-month post-episode period. A p-value less than <0.05 indicates statistical significance. OSC: Other similar CAMPs; PPECM: Porcine placental extracellular matrix.

Patients were assessed in the subsequent 6 months for healthcare utilization in Medicare Parts A and B. The point estimates of the Poisson regression found significant differences in utilization between the three treatment groups (Figure 5). The PPECM compared with the OSC group had significantly less utilization across all care settings. PPECM compared with PPECM’s 510(k) predicate had significantly fewer visits in the physician’s office (27.52, CI: 27.45–27.60, p < 0.0001) and outpatient hospital (5.84, CI: 5.81–5.88, p < 0.0001). Additionally, across the less frequently utilized care sites, PPECM had significantly fewer visits for home health, ICU and DME compared with both comparator treatment groups.

## Discussion

This analysis is among the first to evaluate PPECM compared with other well-established CAMPs in the Medicare population. The results demonstrated comparable outcomes between PPECM and the other treatment groups. After IPTW balancing observable differences between the cohorts, the analyses found a statistically significant lower relative risk of amputation and wound complications in episodes of DFU care that utilized PPECM. Specifically, PPECM episodes were associated with a 30.9% lower risk of outpatient amputation compared with the OSC group (OR: 1.309, p < 0.0001). This was supported by the Cox hazard analysis on the time to first amputation in the 6-month episode post period, which found that the relative risk of amputation was 5.8% higher in OSC episodes (HR: 1.058, CI: 1.022–1.095, p = 0.002) as compared with PPECM episodes. The lower relative risk of amputation could be related to a lower relative risk of potential wound complications such as bacteremia, which was 2.75-fold higher in the OSC group than in the PPECM group. However, given the retrospective nature of this claims data analysis, it is not possible to determine whether these are causally linked. In terms of healthcare utilization, the PPECM group had lower rates of inpatient and ICU visits compared with the OSC group. Utilization rates were similar for PPECM and PPECM’s 510(k) predicate.

The impact of including CAMPs in the treatment of lower extremity diabetic ulcers has been previously examined using CMS claims data by Armstrong *et al.* [23]. In their analysis of the Medicare Limited Data standard analytic hospital inpatient and outpatient department files from 2015 to 2018, the authors demonstrated superior outcomes for CAMPs over standard wound care. Notably, lower extremity diabetic ulcers managed with CAMPs had significant reductions in major and minor amputation rates, emergency visits and hospital readmissions, as compared with those managed with standard wound care. Though it is considered foundational to the current study, the previous examination is not without limitation. The Medicare Limited Dataset only includes claims from inpatient and outpatient settings and does not include claims from physician offices, or other data from Medicare Parts A, B and D. Additionally, propensity score matching was used to generate equivalent cohorts for comparison; while statistically robust, this technique typically results in significant attrition of the starting sample size. Of the nearly 55,000 patients eligible for inclusion in the previous analysis, fewer than half (∼25,000, 45%) were successfully matched and included in the final analysis.

The current study leverages more recent data (2020–2024) from the CMS 100% RIFs database, which encompasses all sites of care for Medicare Parts A and B. Furthermore, the IPTW approach used here allowed for all identified beneficiaries to be used in the analysis, thereby eliminating the potential for attrition seen with matching techniques. The IPTW methodology allows for balanced cohorts, and parallels the effect of randomization in interventional trials [25], which in turn ensures that the observed differences in outcomes have a more direct relationship to potential differences between CAMP treatments. However, additional prospective comparative research would be necessary to confirm these observations.

## Limitations

The study examines source data from one payer (Medicare FFS) and the results of the analysis may not extend to other, younger populations. This study is a retrospective analysis of administrative claims which does not allow for consideration of clinical decisions or uniformity in quality of care by site, patient compliance to care or details that are incomplete from claims data.

## Conclusion

This 4-year retrospective analysis of the Medicare Claims database found that patients treated with PPECM had similar or better clinical outcomes, with a potential reduction in healthcare utilization and fewer visits across all care settings compared with OSC. This evidence builds on prior literature that showed significantly reduced rates of amputations in patients with DFUs managed with a wide variety of CAMPs. These findings may help guide healthcare practitioners and payers in choosing an effective and efficient solution for their patient population.

## Summary points

- Diabetic foot ulcers (DFU) are often hard-to-heal and may require advanced treatment with cellular, acellular and matrix-like products (CAMPs).
- The primary objective of this study was to comparatively evaluate the clinical effectiveness of CAMPs within the 100% Medicare Research Identifiable Files (January 2020 to June 2024).
- Relevant outcomes of interest included rate of amputations, frequency of wound complications, as well as healthcare utilization.
- Patients with diagnoses of DFU and non-pressure chronic ulcers with at least one medical claim in Medicare Parts A and B were selected from the period between 1 January 2020 and 31 December 2023.
- Eligible patients were categorized into groups according to treatment received: porcine, placental extracellular matrix (PPECM), PPECM’s 510(k) predicate or other similar CAMPs (OSC).
- A total of 34,664 patients with DFUs received a CAMP treatment (3.6% of all patients with DFUs).
- Patient demographics, comorbidities and ulcer location were assessed for cohort homogeneity via inverse probability of treatment weighting, allowing for balanced comparison of health outcomes.
- PPECM was associated with a 30.9% lower risk of outpatient amputation compared with the OSC group (OR: 1.309, p < 0.0001).
- This was supported by the Cox-hazard analysis on the time to first amputation in the 6-month episode post period which found that the relative risk of amputation was 5.8% higher in the OSC group (HR: 1.058, CI: 1.022–1.095, p = 0.002) compared with the PPECM group.
- This 4-year retrospective analysis of the Medicare Claims database found that patients treated with PPECM had similar or improved clinical outcomes with potential reduction in healthcare utilization and fewer visits across all care settings compared with OSC.
